# In-Depth Global Analysis of Transcript Abundance Levels in Porcine Alveolar Macrophages Following Infection with Porcine Reproductive and Respiratory Syndrome Virus

**DOI:** 10.1155/2010/864181

**Published:** 2011-01-12

**Authors:** Laura C. Miller, John D. Neill, Gregory P. Harhay, Kelly M. Lager, William W. Laegreid, Marcus E. Kehrli

**Affiliations:** ^1^USDA, Agricultural Research Service, National Animal Disease Center, Virus and Prion Research Unit, 1920 Dayton Ave, Ames, IA 50010, USA; ^2^USDA, Agricultural Research Service, United States Meat Animal Research Center, Animal Health Research Unit, State Spur 18D, Clay Center, NE 68933, USA; ^3^Department of Pathobiology, College of Veterinary Medicine, University of Illinois, 2001 South Lincoln Avenue, Urbana, IL 61802, USA

## Abstract

Porcine reproductive and respiratory syndrome virus (PRRSV) is a major pathogen of swine worldwide and causes considerable economic loss. Identifying specific cell signaling or activation pathways that associate with variation in PRRSV replication and macrophage function may lead to identification of novel gene targets for the control of PRRSV infection. Serial Analysis of Gene Expression (SAGE) was used to create and survey the transcriptome of *in vitro* mock-infected and PRRSV strain VR-2332-infected porcine alveolar macrophages (PAM) at 0, 6, 12, 16, and 24 hours after infection. The transcriptome data indicated changes in transcript abundance occurring in PRRSV-infected PAMs over time after infection with more than 590 unique tags with significantly altered transcript abundance levels identified (*P* < .01). Strikingly, innate immune genes (whose transcript abundances are typically altered in response to other pathogens or insults including IL-8, CCL4, and IL-1*β*) showed no or very little change at any time point following infection.

## 1. Introduction

Porcine Reproductive and Respiratory Syndrome Virus (PRRSV), the causative agent of porcine reproductive and respiratory syndrome (PRRS) in swine, is a member of the *Arteriviridae* family in the order *Nidovirales*. PRRSV causes significant losses to the swine industry worldwide [1] as a result of both reproductive failure (late-term abortions and stillbirths) in pregnant sows and respiratory disease (pneumonia) in nursery and grower/finishing pigs [2]. Infection with PRRSV also predisposes pigs to infection by bacterial pathogens as well as other viral pathogens [3–7]. Clinical disease caused by PRRSV is highly variable, ranging from mild, subclinical infections to acute deaths of swine of any age [8]. Differences in virulence have been attributed to numerous factors including host genetics, management practices, and virus strain heterogeneity [9–16]. 

Relatively little is known about the interactions of PRRSV and host cells. The primary cellular target of PRRSV is the porcine alveolar macrophage (PAM) [17, 18]. PRRSV has been shown to replicate to varying degrees in peritoneal macrophages, pulmonary intravascular macrophages, type II pneumocytes, testicular germ cells, and PAMs [19–22]. MARC-145 cells, a monkey kidney cell line, are used to propagate this fastidious virus in culture. A putative cell surface receptor has been identified that may contribute to the propensity of PRRSV to readily infect these cells. The CD163 protein, sialoadhesin, and heparan sulphate have been reported to play significant role in helping PRRSV attach and be internalized into cells [23].

A primary function of the PAM is to combat bacterial insults within terminal airways of the lung, in part, by regulating the local host immune response in the alveoli. Reports have shown pigs infected with PRRSV have a higher rate of concurrent or secondary bacterial infections [23, 24]. This has led investigators to examine the effect of PRRSV infection on bacterial killing by PAMs [25–28]. Studies have reported PRRSV infection significantly decreases production of superoxide anion and hypohalous acid, both of which contribute to the oxidative ability of PAMs to kill bacteria [25]. Another report [29] demonstrated that PRRSV infection resulted in cytotoxicity to PAMs that led to a 40% reduction in phagocytic uptake of *Escherichia coli*. The specific mechanism(s) by which PRRSV infection alters PAM function is unknown. It has been reported PRRSV infection of PAMs results in lowered transcript abundance of proinflammatory response cytokines including TNF-*α*, IL-1*α*, and MIP-1*β* [30]. However, other studies showed that TNF-*α*, IL-8, IFN-*α*, and IL-1*β* transcripts are not significantly altered by PRRSV infection [31–35]. Thus, it remains unclear which macrophage genes PRRSV affects upon infection. 

 One study attempting to better understand the altered transcript abundance of PAMs upon infection by PRRSV used differential display reverse-transcription PCR to identify host cell gene responses to PRRSV infection of PAMs over a 24 hour period [35]. Four transcripts were identified that specifically responded to PRRSV infection and were induced *in vivo* in tissues where PRRSV persistently resides. Of the four transcripts identified, three of these came from identified genes and the fourth remains a novel expressed sequence tag (EST). The three genes identified are Mx1 (myxovirus resistance), UBP (ubiquitin protease), and RHIV-1 (RNA helicase). Presumably there are more, yet to be identified, genes that differentially respond to PRRSV infection. 

Various research techniques provide the potential to look at cellular processes and response to infection in a comprehensive and unbiased manner [36]. Combined with targeted approaches such as gene knockouts, transgenics, targeted overexpression and other methods, it is now possible to dissect pathways and networks of genes, proteins and small molecules that define cellular functions. Technologies have been developed that permit high throughput quantification of transcript abundance. Most prominent of these are various forms of solid phase microarray hybridization [37] and serial analysis of gene expression (SAGE; [38, 39]). Information derived from these methods can be exploited in a number of ways including development of diagnostic assays, understanding molecular mechanism(s) behind disease states and formulating intervention regimens to inhibit or minimize infections and negative outcomes. SAGE has been used extensively to evaluate changes in transcript abundance in a number of experimental systems. The availability of nearly 600,000 swine and over 1 million bovine ESTs in the NCBI dbEST greatly increases the utility of SAGE for gene expression studies in these species. SAGE has also been used for gene discovery and enabled identification of genes not previously known to be expressed in granulocytes [40], and in identifying potential new cell surface diagnostic markers in astrocytomas [41]. 

It is our hypothesis that PRRSV infection of PAMs alters their normal transcriptome in a manner that enables virus replication and dysregulates the normal host immune response. Here we report a comprehensive evaluation of transcript abundance levels in noninfected and PRRSV-infected PAMs as an initial step towards a more comprehensive understanding of PRRSV pathogenesis. Although a comprehensive understanding of differential posttranscriptional and posttranslational responses in PAMs remains to be determined, detection of altered transcriptome patterns may identify PRRSV virulence mechanisms that contribute to a delayed or lack of a protective immune response and viral persistence.

## 2. Results

### 2.1. Serial Analysis of Gene Expression

Total cellular RNA was prepared from *in vitro* PRRSV-infected PAMs at 0, 6, 12, 16 and 24 hours after infection, and mock-infected PAMs at 0 and 24 hours. Five SAGE libraries were constructed from the 0 hour mock-infected and the 6, 12, 16 and 24 hours PRRSV-infected cells. The libraries were subsequently sequenced to obtain approximately 100,000 tags each, with the exception of the 24 hour infected library, which was sequenced to nearly 200,000 tags (Table 1). Five SAGE libraries yielded 643,255 sequenced tags that were used to populate a modified Identitag database. Examination of the SAGE data indicated that there were major changes in transcript abundance occurring in the PRRSV-infected PAMs based on more than 590 unique tags with significantly altered transcript abundance (*P* < .001 with Bonferroni correction). Table 1 summarizes the general statistics of these libraries. Tags with a frequency of 1 were not considered for quantitative purposes, because they could represent artifacts of sequencing or of the SAGE procedure [42].

### 2.2. Functional Classification of Transcripts with Changes in Abundance

To obtain a greater understanding of cellular responses to PRRSV, the identified transcripts were further categorized with biological processes, defined by the Gene Ontology Consortium (http://www.geneontology.org/), according to the Ingenuity Pathway Analysis (IPA) database (Ingenuity Systems, http://www.ingenuity.com/). Fischer's exact test was used to calculate a *P*-value determining the probability that each biological function assigned to that data set is due to chance alone. Transcriptional abundance differences between high-level function groups observed during the first 24 hours of PRRSV infection were compared. Cellular movement, which describes the cellular functions associated with movement and localization of cells, was significantly upregulated among differentially expressed transcripts in the first 6 h p.i. Cell-to-cell signalling and interaction, which describes functions involved in intercellular interactions and includes functions associated with specific cellular components that are involved in signalling and interaction, an important aspect of macrophage function, included the most significantly down-regulated, differentially expressed transcripts. Our interest was in those expression changes that affect PAM function; particularly in regard to innate immunity, antigen presentation, and intra- and extra-cellular signalling.

### 2.3. Detection of Viral RNA Transcripts

Analysis of the data revealed the presence of the tags derived from transcripts produced by the infecting PRRSV strain; thus confirming the virus infected the PAMs as intended. PRRSV produces 3′ co-terminal subgenomic RNAs, thus all viral RNAs had the same tag [43]. The viral tag, not detected in the mock-infected library, was first present at 6-hours PI and increased in number to its highest point at 12 hours after which it declined (Table 1). These data were validated by real-time PCR (data not shown). There was a high level of viral RNAs present in the infected cells at 12 hours after infection, where viral RNAs accounted for almost 10% of all polyadenylated RNA in these cells. The amount of viral RNA declined by 24 h hours after infection perhaps because of a decline in RNA replication/transcription, degradation of viral RNAs, release of virus from the cells or a combination of all three.

### 2.4. Real-Time PCR Validation of Transcript Abundance Levels

**Figure 1:**
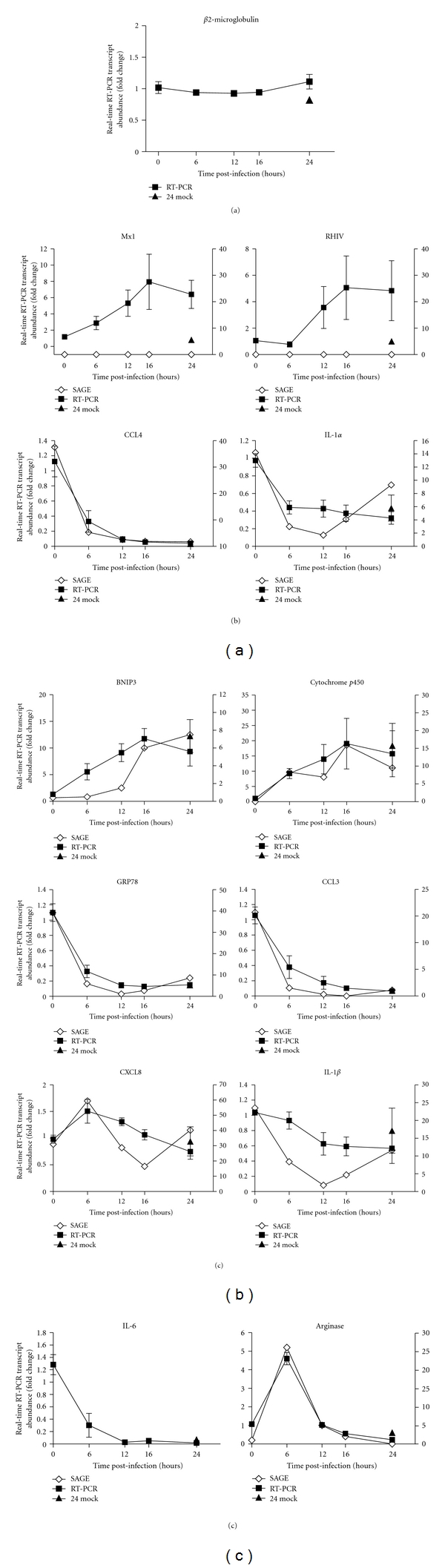
Real-time RT-PCR validation of SAGE results. Validation by real-time RT-PCR on RNA from the *in vitro* PRRSV-infected PAMs samples used in the SAGE analysis of selected transcripts showing differential transcript abundance. Real-time RT-PCR transcript abundance results (filled squares; left *y*-axis) expressed as the mean fold increase ± SEM in gene expression (*n* = 3) relative to mock-infected cells and SAGE tag counts normalized to total tags per library (open diamonds; right *y*-axis) are shown for transcripts. The “24 mock” result shows the real-time RT-PCR fold change in transcript abundance as a result of being in cell culture.

Changes in transcript abundance of genes of interest were validated using real-time reverse transcriptase (RT)-PCR (Figure 1). In this study, *β*2-microglobulin was found to have stable transcript levels across all times tested (Figure 1(a)) and was chosen as the internal control in all real-time RT-PCR assays. 

Real-time RT-PCR amplification of cellular transcripts previously shown to have altered transcript abundance levels following PRRSV infection was done to confirm the infection had proceeded as expected (Figure 1(b)). As expected, transcripts encoding the proteins Mx1 and rHIV (a RNA helicase) showed 6.4- and 4.8-fold increases at 24 hours PI, respectively (Figure 1(b)), and were induced between 0 and 12 h postPRRSV-infection [35]. The transcripts encoding the proinflammatory proteins IL-1*α* and CCL4 (macrophage inflammatory protein, MIP-1*β*) declined in abundance as previously demonstrated [44]. The tags for the Mx1 transcript were identified in the 16 and 24 hour infected SAGE libraries but at very low levels and were therefore insignificant following normalization of the SAGE data.

Overall, the real-time RT-PCR validation described above, in addition to that which was not shown, had a high degree of correlation to the SAGE data, thus providing confidence that the tag counts for each library was an accurate representation of transcript abundance levels in these PAMs. This demonstrated that additional validation of the SAGE libraries by real-time PCR was not necessary.

### 2.5. Transcripts Encoding Immune Response Proteins

There was a general decline in numbers of the cytokine and chemokine transcripts, indicating that there was no induction of expression that would be expected in an innate immune response to a viral infection. SAGE revealed decreased transcript abundance IL-1*α* and IL-1*β* (Tables 2(a) and 2(b)). RANTES, MIF, MCP3 and CXCL5, showed increased transcription during the infection (Table 2(a)). MCP3 and CXCL5 showed increases only late in the infection process, while CXCL8 (IL-8) showed increased transcription early in the infection then decayed to noninfected levels (Table 2(a)). Interestingly, there were 2 tags identified for CXCL8 with both showing similar patterns of expression. Proinflammatory cytokines CXCL8 and CXCL5 are chemoattractants for neutophils, IL-1*α* and IL-1*β* are important proinflammatory cytokines and would be expected to be increased early in infection. IL-1*α* and IL-1*β* promote infiltration of various leukocytes by inducing chemokine production and increasing expression of various adhesion molecules on mesenchymal cells and postcapillary venule endothelial cells.

The increase in transcript numbers of cytochrome p450 3A29, BNIP3 (a proapoptotic protein), and GRP78 (an endoplasmic reticulum chaperone protein) were also confirmed (Figure 1(c)). Changes in levels of transcripts encoding proteins involved in defense and the innate immune response were also validated (Figure 1(c), Table 2). Changes in levels of transcripts encoding CCL3 (MIP1-*α*), CXCL8 (swine IL-8), and IL-1*β* were found to be very close to that indicated by SAGE with all declining as the infection progressed. An additional transcript of interest, that encoding IL-6, was not found represented in the SAGE database. Interestingly, the transcript encoding IL-6 does not encode a tag (no *Nla* III restriction site in the transcript), therefore, it was not detected by SAGE. The only information on IL-6 transcript abundance levels in these cells was obtained by real-time RT-PCR. This revealed a sharp decline in IL-6 transcripts during the first 24 hours following infection by PRRSV (Figure 1(c)).

### 2.6. Additional Pathways and Functions

Data was evaluated for indications of activation of intracellular signaling or other pathways in PRRSV-infected PAMs. Tags derived from transcripts encoding major proteins making up the AP-1 immediate-early transcriptional complex were identified (Table 2(c)). Tags corresponding to the *c-jun*, *junB, junD* and *c-fos* transcripts showed decreased abundance levels, dropping to the lowest levels at 12 hours and showing some rebound by 24 hours PI. These data indicated that there was no overt induction of the AP-1 signaling pathway following PRRSV-infection. Similarly, activation of NF-*κ*B was not observed (Table 2(c)). A20, a negative regulator of NF-*κ*B as well as a gene that is transcriptionally activated by NF-*κ*B, showed no significant changes in the SAGE data. Interestingly, I*κ*B*α* showed a steady decline in transcript abundance out to 24 hours PI while I*κ*K*α* showed no significant changes over the course of the experiment. Activation of toll-like receptor 4 (TLR4), a cell surface receptor that recognizes bacterial cell membrane lipopolysaccharides, results in the downstream activation of NF-*κ*B. The transcript encoding TLR4 showed a steady decline during the course of the PRRSV infection (Table 2(c)). 

An interesting finding was the sharp increase in arginase transcript levels at 6 hours PI, with the RT-PCR and SAGE data closely mimicking each other (Figure 1(c)). In addition, a total of four distinct tags, presumably representing different mRNA species, were found that were derived from arginase-encoding transcripts; all showed increased transcript abundance at 6 hours PI (Table 2(c)). The primer set used to validate the arginase transcript levels amplified sequences found within the coding sequences of the transcript, thus, the real-time PCR results were from amplification of sequences from all four transcripts. The 6 hour PI time point examined here represents only a “snap-shot” of the cells at that time, so the full pattern of expression in not known.

## 3. Discussion

The derived catalog of expressed genes reported here represents a first attempt to generate comprehensive analyses of PRRSV-infected PAMs transcript abundance profiles at different time points after infection. The wealth of information obtained allows detection of altered transcription of genes involved in normal porcine alveolar macrophage physiology, as well as genes whose transcript abundance is altered by PRRSV infection.

The ability of an animal to respond to specific foreign characteristics or molecular patterns of pathogens is an important aspect of innate immunity. This is the first, generally rapid step in the response to invasion by a pathogen and the beginning of a protective immune response. It is important that this response begin quickly and in sufficient fashion to stop the spread of the invader and terminate the infection. In many cases, the pathogen possesses the ability to inhibit or thwart the immediate innate immune response, thus giving it an early advantage. Deciphering the mechanisms of disruption of the innate immune response is the focus of considerable research and is beginning to provide answers into the myriad of different means pathogens employ to achieve this. The focus here was to discern how PRRSV inhibits the innate immune response in infected PAMs. This study produced transcriptional profiles of noninfected and PRRSV-infected PAMs that provided insight into the suppression by the virus on host transcript abundance levels necessary for a strong immune response. This work has resulted in the characterization of macrophage transcript abundance in normal cells as well as transcript abundance changes that occurred with progressive PRRSV replication. Virus-specific transcript abundance changes that were found provided intriguing clues to possible mechanisms behind immune suppression and the lack of a strong innate and adaptive immune responses. Functional genomic analyses of pure populations of PAMs freshly obtained from healthy pigs revealed that PRRS virus fails to elicit a significant (>2-fold) increase in the transcription of immune-related genes. The character of the innate immune response to a virus is thought to dictate the quality of the adaptive immune response that ensues. Given the key roles that the cytokines produced by cells of the innate immune system, play in the development of adaptive immunity, clarification of the pathways responsible for modulating their generation during the initial innate immune response to PRRSV could have important implications in the development of effective vaccines against this major pathogen of swine. The results obtained in this project helped us understand that the unique character of the innate immune response to PRRS virus is likely to be influencing the quality of adaptive (acquired) immune response to this virus. Thus, the knowledge derived from this study will contribute to the elucidation of the molecular mechanisms controlling the development of adaptive immunity in swine to PRRS virus and will allow for the rational development of effective vaccines against this pathogen.

Previous work has demonstrated transcript abundance differences in 13 genes between noninfected and PRRSV-infected PAMs [33, 35, 45, 46]. More recently, Genini et al. [47] profiled gene expression of PAMs infected in vitro with the European Lelystad strain of PRRSV, with the biological similarities but distinct serological properties from the North American VR-2332 isolate [48], over 12 h p.i. by utilizing an Affymetrix 24 K Porcine Chip array. In Genini's study, statistical analysis of variance (ANOVA) showed differential expression of 1409 genes. After applying a cut-off threshold based on a fold-change of 1.5 between infected and control PAMs, 148 genes were differentially expressed compared with the controls [47]. In our study, greater than 590 significant (*P* < .01) changes in transcript abundance levels were identified. 

Analysis of this transcriptomics data in the context of gene ontology allows us to ascribe biological function to the differentiated transcript abundance dataset. The *P*-values and scores calculated in IPA act as starting points for further investigation and act as rough guides for identification of significant processes or pathways affected in the experiment. Note, however, that functions whose significance values exhibit little or no change from one time-point to another may be changing. 

However, an important aspect of this study was not in what was altered, but rather what was not. Of particular interest was the apparent lack of any overt innate immune response in the PRRSV-infected PAMs. This was borne out by the lack of increased transcription of chemokine and cytokine genes that are commonly observed to increase in infection with other pathogens. These included CCL3, CCL4, TNF*α*, type 1 interferons, and a number of proinflammatory chemokines and cytokines.

In the initial stage of a PRRSV infection, the primary target cells of the virus are PAMs. It is as yet unknown what signals are necessary to call the immune system into action. The most likely candidates are cytokines and particularly those that initiate migration and activation of leukocytes. Sprenger et al. [49] have shown that influenza A virus selectively induces mononuclear leukocyte-attracting chemokines MIP-1, MCP-1, and RANTES and suppresses neutrophil-attracting chemokines IL-8 and GRO-*α*. In this study, we have shown that PPRSV does not activate an alveolar macrophage proinflammatory response while suppressing type 1 IFN production and apoptotic pathways. No activation of the PAMs was indicated by a decrease in IL-1*α*, IL-6, or IL-8. Knoetig et al. [50] has shown that IL-1 is released from CSFV-infected macrophages. IL-8 is, for example, an important chemo-attractant for immune cells, while IL-1 and IL-6 prime B- and T-cell responses against infected cells. The chemokines CCL3 (MIP-1*α*), CCL4 (MIP-1*β*), RANTES, MCP1, MCP3, and MIP3 are important chemo-attractants and mediators of virus-induced inflammation *in vivo* [51]. Cytokine mRNAs have a short half-life after synthesis and the rapid reduction in mRNA levels a few hours after addition of virus suggests rapid intracellular cytokine mRNA degradation or suppressed transcription. Virus replication and protein synthesis has been shown to commence 10–15 h PI [52] (although SAGE showed an increase in viral RNAs at 6 hours PI), suggesting that any increase in cytokine mRNA levels after 15 h PI was due to the presence of replicating virus. Similarly, we showed no activation of NF-*κ*B, indicating a lack of transcriptional activation of these genes. The transcription factor NF-*κ*B is a central regulator of the transcript abundance of these proinflammatory genes and many viruses manipulate the NF-*κ*B pathway, resulting in suppression of antiviral responses or prevention of apoptosis [53].

The finding of a sharp spike in transcription of the arginase gene at 6 hours PI may give a clue to the early events following infection that inhibit an innate immune response. Arginase competes with nitric oxide synthases (NOS) for the substrate arginine for the production of nitric oxide (NO). NO is an important early signal in many pathophysiologic processes. Early inhibition of its production would have an impact on downstream events. Increased transcript abundance of arginase has been shown to modulate NO production in macrophages and impact the downstream immune response [54–56].

## 4. Conclusions

It is well established that many pathogens cause changes in expression of specific genes that act to protect the host and clear the infection. This type of response was not seen in these PRRSV-infected PAMs. Of particular interest was the minimal expression of genes that are involved in attracting other immune cells to the area of the infection. Additionally, there was no response by genes that cause inflammation. This is the first comprehensive study to show the inhibition of an immune response in PAMs by PRRSV. However, the results have also given us tantalizing clues to the mechanism(s) behind this inhibition. There are specific cellular proteins that control the expression of the protective genes and future studies of the genes, their transcript abundance, protein level, and protein function will enhance our understanding of the interaction of PRRSV with the porcine macrophage. Possible outcomes may include identification of virulence mechanisms, development of diagnostic assays and rational vaccine design to more effectively limit viral replication and spread.

## 5. Methods

### 5.1. Cells and Virus

Primary PAMs were isolated, cultured, and infected, as previously described [57]. Briefly, PAMs were harvested from three clinically healthy, PRRS-negative gilts 6–8 weeks of age. Animals were humanely euthanized, following animal care and use protocols, and PAMs were harvested under aseptic conditions. PAMs were tested by PCR for porcine circovirus and *Mycoplasma* spp [58, 59] and found to be free of both. Aliquots of PAMs were frozen and stored in liquid nitrogen. Typical yields were 10^8^–10^9^ PAMs with >95% viability. Immediately prior to use, PAMs were thawed and viability of PAMs was determined to be 85% to 90% by trypan blue dye exclusion. Viable PAMs were cultured at 37°C, 5% CO_2_ in Dulbecco's Modified Eagles Media with 5% fetal bovine serum (FBS; Gibco-Invitrogen, Carlsbad, CA) and 1% antibiotic/antimyotic (Gibco-Invitrogen) for 2 hours. PRRSV strain VR-2332 [60] stock was propagated in MARC-145 cells [61] and stored frozen at −80°C until use.

### 5.2. Infection and RNA Isolation

PAMs isolated from three pigs were maintained separately. All three sets of PAMs were treated identically. After establishing PAMs in culture, cells were infected with PRRSV strain VR-2332. To achieve a near synchronous infection, flasks containing adherent PAMs were infected at a multiplicity of infection (MOI) of 10 in chilled media and incubated at 4°C for 1 hour to allow for virus binding, but not entry into the cell. Pre-warmed media was added and the cells placed at 37°C, 5% CO_2_ until collected for RNA isolation. Total cellular RNA was prepared using the Qiagen RNeasy mini kit (Qiagen, Valencia, CA), according to manufacturers instructions, from each PRRSV-infected PAMs flask at 0, 6, 12, 16 or 24 hours after infection. Total cellular RNA from mock-infected PAMs was collected at 0 and 24 hours.

### 5.3. SAGE Library Construction

SAGE libraries were constructed as described previously [62] using *Nla* III as the anchoring enzyme. Each library was made from pooled equimolar amounts of total RNA from each pig at each time point. The SAGE libraries provided the population means of the transcript abundance levels for each time point. SAGE clones were amplified and sequenced using a high-throughput sequencing pipeline with an ABI 3730 automated sequencer and ABI chemistry (Applied Biosystems Inc., Foster City, CA). The SAGE libraries with tag counts were submitted to GenBank GEO and have the accession number GSE10346. 

The database of tags derived from the raw sequence data was analyzed to identity transcripts from which tags were derived as well as their relative abundance. Tag sequences were corrected for sequencing errors using R and sagenhaft [63]. The libraries were normalized to total tags. Relative abundance was calculated based upon the number of times a tag was represented in a given SAGE library [64]. Tags were mapped to transcripts and genes by exact regular expression matching to sequences in GenBank, Harvard Gene Index, and the Pig Expression Database (Japan) databases and parsed into a modified Identitag database [65]. Multidimensional statistical tests: Audic and Claverie pairwise test; the Fisher's exact test; Greller and Tobin test; the R test and pairwise and general Chi-square tests [66] were applied to determine which changes in tag abundance were significant. The Fisher's exact test was adequate for detecting differences in gene expression when dealing with pairwise comparisons. SAGE libraries were compared with each other to identify common or differential patterns of transcript abundance. Attention was given to those transcripts where transcript abundance changes may affect PAM function; particularly in regard to innate immunity, antigen presentation, and intra- and extracellular signaling.

### 5.4. Real-Time RT-PCR Validation

Validation of the results and corroboration of the altered transcript abundance levels were analyzed by real-time reverse transcription-PCR (RT-PCR) on the individual sample of 100 ng total RNA from each pig at each time point. Real-time RT-PCR was done in 25 *μ*L reaction volumes using the SuperScript III Platinum SYBR green One Step qRT-PCR kit (Invitrogen, Carlsbad, CA) according to the supplier's specifications. The primer sets used for this analysis are shown in Table 1. All primers were used at 200 nM. PCR cycling conditions were 95°C for 15 minutes followed by 40 cycles of 94°C for 10 seconds, 60°C for 30 seconds and 72°C for 30 seconds using an Opticon 2 fluorescent thermocycler (BioRad, Inc., Hercules, CA). Final analysis of amplification products was done by melt curve where the PCR reactions were heated from 50 to 94°C at a rate of 0.5°C /second. Equal amplification kinetics of the target and the reference genes (*β*2-microglobulin) were confirmed by serial dilutions as described [67]. Quantification of levels of mRNA were calculated using the 2^−ΔΔCt^ method, which expresses mRNA in treated cells relative to mock infected cells after normalizing to *β*2-microglobulin (*β*2m) [68]. For real-time PCR and SAGE tag count comparisons, *β*2-microglobulin (*β*2m) served as the internal control where the number of SAGE tags for *β*2m derived from this library and the amplification curve from real-time PCR were considered equal.

##  Authors' Contributions 

L. C. Miller: study conception, data collection and analysis, research design, manuscript writing. J. D. Neill: study conception, research design, SAGE library construction, manuscript production. G. P. Harhay: research design, SAGE database construction, data analysis, manuscript production. K. M. Lager: virus stocks, data collection, manuscript production. W. W. Laegreid: study conception, manuscript production. M. E. Kehrli: study conception, manuscript production. All authors read and approved the final manuscript.

##  Disclaimer 

Mention of trade names or commercial products in this article is solely for the purpose of providing specific information and does not imply recommendation or endorsement by the U.S. Department of Agriculture. USDA is an equal opportunity provider and employer.

## Figures and Tables

**Table 1:** Summary statistics of SAGE libraries.

Library (hours PI)	Noninfected	Infected			
	0	6	12	16	24
Tags sequenced	111,214	96,968	103,662	134,990	196,421
Unique tags	24,367	30,709	33,601	33,255	37,942
PRRSV tag (CGGCCGAAAT)	0	255	9500	6902	3632

**Table:** (a) Changes in chemokine transcript abundance in PRRSV-infected PAMs

Transcript	Function	RT-PCR	Normalized SAGE tag counts (per million tags)				
			Mock	6	12	16	24
CCL3 (MIP1*α*)	Chemotaxis	↓	191	21	3	0	21
CCL4 (MIP1*β*)	Chemotaxis	↓	419	68	25	19	16
CCL5 (RANTES)	Chemotaxis	nd	7	7	17	23	13
MIF	Inhibits macrophage migration	nd	8	1	25	35	32
CCL20 (MIP3*α*)	Chemotaxis	nd	196	78	14	19	11
CCL2 (MCP-1)	Chemotaxis	nd	1	1	1	4	31
CCL7 (MCP-3)	Chemotaxis	nd	0	0	4	1	22
CXCL2 (GRO*β*)	Chemotaxis	↓	1596	659	335	215	155
CXCL8 (AMCF-1/IL-8)*	Chemotaxis	6 hr ↑	353 8	807 172	370 121	202 55	449 17
CXCL5 (AMCF-2)	Chemotaxis	6 hr ↑	0	88	31	13	80

**Table:** (b) Changes in cytokine transcript abundance in PRRSV-infected PAMs

Transcript	Function	RT-PCR	Normalized SAGE tag counts (per million tags)				
			Mock	6	12	16	24

IL-6	Acute phase response, T- and B-cell growth & differentiation	↓	No tag in transcript				
GM-CSF	Dendritic cell growth & differentiation	nd	4	3	1	0	4
TNF-*α*	Local inflammation, endothelial activation	nd	49	3	1	0	0
IL-1*α*	T-cell activation, macrophage activation	↓	152	31	23	16	40
Il-1*β*	T-cell activation, macrophage activation	↓	283	103	31	52	163

**Table:** (c) Changes in Immediate Early-Response Transcript Abundance in PRRSV-infected PAMs

Transcript	Function	RT-PCR	Normalized SAGE tag counts (per million tags)				
			Mock	6	12	16	24

AP-1	Transcription factor	nd	1	0	0	0	0
C-jun	Transcription factor for AP-1	nd	8	3	0	1	5
C-fos	Transactivating regulator of gene expression	nd	134	9	11	18	9
JunB	Transcription factor for AP-1	nd	101	6	11	17	33
JunD	Transcription factor for AP-1	nd	13	1	0	4	9
NF*κ*B	Transcription factor	nd	2	1	3	0	0
I*κ*B*α*	NF*κ*B inhibitor	nd	205	72	42	11	26
I*κ*K*α*	Dissociates inhibitor from NF*κ*B	nd	4	7	3	2	1
A20	Inhibits activation of transcription factors	nd	11	12	6	1	4
TLR4	Activation of transcription factor NF*κ*B	nd	24	22	14	4	15
Arginase*	Regulator of nitric oxide synthesis	6 hr ↑	227 37 1 14	915 131 88 84	169 21 17 31	141 18 4 5	103 24 1 3

*Alternative spliced transcripts.

**Table 3:** Real-time PCR primer sequences.

Transcript	Sequence ^(1)^
*β*2-microglobulin	GCAGTCAGACCTGTCTTTCAGCAA ATCTCTGTGATGCCGGTTAGTGGT
*β*-actin	AGATGTGGATCAGCAAGCAGGAGT AGCTAACAGTCCGCCTAGAAGCAT
A20	GATGCTGCCTTTGATGCTGGTCTT AAAGCACACAGAGACACTGCAAGC
Arginase	AAGAACGGAAGGACCAGCCTTGTA TCGTGGTTGTCAGTGGAGTGTTGA
BNIP3	TTCGCGTCTCCTGAATCACCTGTA AGTGCCTAACTCAAGGCTGCAGAT
CCL3 (MIP-1*α*)	AAACAGCCACTCTCTGGGACTCAT AAAGGTGTCTTCGGACCTCTTGGA
CCL4 (MIP-1*β*)	AAGCTTCCTCGCAACTTCGTGACT TCAGAGCAGCTCAGTTCAGTTCCA
CD163	TCTGTTGGCCTGTCTCATCGCATT TCAGGCAAGAATTCATCTCCCGGT
CXCL2	GACCGTGCAAGGAATTCACCTCAA CAGTTGGCACTGCTCTTGTTTAGC
CXCL5(AMCF2)	AGCCACCCTGAAGAATGGAAAGGA CTTCTGCTGAAGAACTGGGCGATT
CXCL8 (IL-8/AMCF1)	GCAGAACTTCGATGCCAGTGCATA TCTGTACAACCTTCTGCACCCACT
Cytochrome P450 3A29	TACCTACGATGGTCTGGCGCAAAT CGAACACGCCATGGATTTCCACAT
GRP78^(2)^	TGGCATTCTTCGAGTGACTGCTGA GTGTCAATGCGCTCCTTGAGCTTT
IL-1*α*	TTCAAATCAGCCGCCCATCCAAAG TGGTACATACGGCCTGTCAACACT
IL-1*β*	GAAATGGGAGCATCCAGCTGCAAA TTGCACGTTTCAAGGATGATGGGC
IL-6	ATGCTCTTCACCTCTCCGGACAAA TTCTGCCAGTACCTCCTTGCTGTT
MIF	TACTACGACATGAACGCGGCCAAT GCGCCATCTCCACACCGTTTATTT
Mx1	TTCGCACATCCTCCTGTGGTTAGT GCGTGCTTATCACAGCTTCTTGCT
PRRSV	CAACGGCAAGCAGCAGAAGAGAAA TGATCTTACCCAGCATCTGGCACA
RHIV	TTTGGACTCTGTTCTCAGGCAGGT AGACTTAAACCCGAGCCTCAGCAA

^(1)^First line of sequence is plus sense and second line is minus sense.

^(2)^Glucose responsive protein 78.

## References

[1] Rossow KD (1998). Porcine reproductive and respiratory syndrome. *Veterinary Pathology*.

[2] Zimmerman JJ, Yoon KJ, Wills RW, Swenson SL (1997). General overview of PRRSV: a perspective from the United States. *Veterinary Microbiology*.

[3] Done SH, Paton DJ (1995). Porcine reproductive and respiratory syndrome: clinical disease, pathology and immunosuppression. *Veterinary Record*.

[4] Feng WH, Laster SM, Tompkins M (2001). In utero infection by porcine reproductive and respiratory syndrome virus is sufficient to increase susceptibility of piglets to challenge by Streptococcus suis type II. *Journal of Virology*.

[5] Rossow KD, Collins JE, Goyal SM, Nelson EA, Christopher-Hennings J, Benfield DA (1995). Pathogenesis of porcine reproductive and respiratory syndrome virus infection in gnotobiotic pigs. *Veterinary Pathology*.

[6] Wills RW, Gray JT, Fedorka-Cray PJ, Yoon K-J, Ladely S, Zimmerman JJ (2000). Synergism between porcine reproductive and respiratory syndrome virus (PRRSV) and Salmonella choleraesuis in swine. *Veterinary Microbiology*.

[7] Zeman D, Neiger R, Yaeger M (1993). Laboratory investigation of PRRS virus infection in three swine herds. *Journal of Veterinary Diagnostic Investigation*.

[8] Mengeling WL, Lager KM (2000). A brief review of procedures and potential problems associated with the diagnosis of porcine reproductive and respiratory syndrome. *Veterinary Research*.

[9] Goldberg TL, Hahn EC, Weigel RM, Scherba G (2000). Genetic, geographical and temporal variation of porcine reproductive and respiratory syndrome virus in Illinois. *Journal of General Virology*.

[10] Goldberg TL, Weigel RM, Hahn EC, Scherba G (2000). Associations between genetics, farm characteristics and clinical disease in field outbreaks of porcine reproductive and respiratory syndrome virus. *Preventive Veterinary Medicine*.

[11] Halbur PG, Paul PS, Frey ML (1995). Comparison of the pathogenicity of two US porcine reproductive and respiratory syndrome virus isolates with that of the Lelystad virus. *Veterinary Pathology*.

[12] Halbur PG, Paul PS, Meng XJ, Lum MA, Andrews JJ, Rathje JA (1996). Comparative pathogenicity of nine US porcine reproductive and respiratory syndrome virus (PRRSV) isolates in a five-week-old cesarean-derived, colostrum-deprived pig model. *Journal of Veterinary Diagnostic Investigation*.

[13] Keffaber KK (1989). Reproductive failure of unknown etiology. * American Association of Swine Practitioners Newsletter*.

[14] Lewis CRG, Ait-Ali T, Clapperton M, Archibald AL, Bishop S (2007). Genetic perspectives on host responses to porcine reproductive and respiratory syndrome (PRRS). *Viral Immunology*.

[15] Meng XJ (2000). Heterogeneity of porcine reproductive and respiratory syndrome virus: implications for current vaccine efficacy and future vaccine development. *Veterinary Microbiology*.

[16] Wensvoort G (1993). Lelystad virus and the porcine epidemic abortion and respiratory syndrome. *Veterinary Research*.

[17] Duan X, Nauwynck HJ, Pensaert MB (1997). Effects of origin and state of differentiation and activation of monocytes/macrophages on their susceptibility to porcine reproductive and respiratory syndrome virus (PRRSV). *Archives of Virology*.

[18] Lawson SR, Rossow KD, Collins JE, Benfield DA, Rowland RRR (1997). Porcine reproductive and respiratory syndrome virus infection of gnotobiotic pigs: sites of virus replication and co-localization with MAC-387 staining at 21 days post-infection. *Virus Research*.

[19] Haynes JS, Halbur PG, Sirinarumitr T, Paul PS, Meng XJ, Huffman EL (1997). Temporal and morphologic characterization of the distribution of porcine reproductive and respiratory syndrome virus (PRRSV) by in situ hybridization in pigs infected with isolates of PRRSV that differ in virulence. *Veterinary Pathology*.

[20] Rossow KD, Benfield DA, Goyal SM, Nelson EA, Christopher-Hennings J, Collins JE (1996). Chronological immunohistochemical detection and localization of porcine reproductive and respiratory syndrome virus in gnotobiotic pigs. *Veterinary Pathology*.

[21] Sur JH, Doster AR, Christian JS (1997). Porcine reproductive and respiratory syndrome virus replicates in testicular germ cells, alters spermatogenesis, and induces germ cell death by apoptosis. *Journal of Virology*.

[22] Sur JH, Cooper VL, Galeota JA, Hesse RA, Doster AR, Osorio FA (1996). In vivo detection of porcine reproductive and respiratory syndrome virus RNA by in situ hybridization at different times postinfection. *Journal of Clinical Microbiology*.

[23] Van Reeth K, Adair B (1997). Macrophages and respiratory viruses. *Pathologie Biologie*.

[24] Zimmerman JJ, Benfield DA, Murtaugh MP, Osario FA, Stevenson GW, Torremorell M, Straw BB, Zimmerman JJ, D'Allaire SD, Taylor DJ (2006). Porcine reproductive and respiratory syndrome virus (porcine arterivirus). *Diseases of Swine*.

[25] Chiou MT, Jeng CR, Chueh LL, Cheng CH, Pang VF (2000). Effects of porcine reproductive and respiratory syndrome virus (isolate tw91) on porcine alveolar macrophages in vitro. *Veterinary Microbiology*.

[26] Solano GI, Segalés J, Collins JE, Molitor TW, Pijoan C (1997). Porcine reproductive and respiratory syndrome virus (PRRSv) interaction with Haemophilus parasuis. *Veterinary Microbiology*.

[27] Thanawongnuwech R, Thacker EL, Halbur PG (1997). Effect of porcine reproductive and respiratory syndrome virus (PRRSV) (isolate ATCC VR-2385) infection on bactericidal activity of porcine pulmonary intravascular macrophages (PIMS): in vitro comparisons with pulmonary alveolar macrophages (PAMS). *Veterinary Immunology and Immunopathology*.

[28] Thanawongnuwech R, Thacker EL, Halbur PG (1998). Influence of pig age on virus titer and bactericidal activity of porcine reproductive and respiratory syndrome virus (PRRSV)-infected pulmonary intravascular macrophages (PIMs). *Veterinary Microbiology*.

[29] Oleksiewicz MB, Nielsen J (1999). Effect of porcine reproductive and respiratory syndrome virus (PRRSV) on alveolar lung macrophage survival and function. *Veterinary Microbiology*.

[30] López Fuertes L, Doménech N, Alvarez B (1999). Analysis of cellular immune response in pigs recovered from porcine respiratory and reproductive syndrome infection. *Virus Research*.

[31] Ait-Ali T, Wilson AD, Westcott DG (2007). Innate immune responses to replication of porcine reproductive and respiratory syndrome virus in isolated swine alveolar macrophages. *Viral Immunology*.

[32] Buddaert W, Van Reeth K, Pensaert M (1998). In vivo and in vitro interferon (IFN) studies with the porcine reproductive and respiratory syndrome virus (PRRSV). *Advances in Experimental Medicine and Biology*.

[33] Choi C, Cho WS, Kim B, Chae C (2002). Expression of interferon-gamma and tumour necrosis factor-alpha in pigs experimentally infected with porcine reproductive and respiratory syndrome virus (PRRSV). *Journal of Comparative Pathology*.

[34] Thanawongnuwech R, Young TF, Thacker BJ, Thacker EL (2001). Differential production of proinflammatory cytokines: in vitro PRRSV and Mycoplasma hyopneumoniae co-infection model. *Veterinary Immunology and Immunopathology*.

[35] Zhang X, Shin J, Molitor TW, Schook LB, Rutherford MS (1999). Molecular responses of macrophages to porcine reproductive and respiratory syndrome virus infection. *Virology*.

[36] Joyce AR, Palsson BØ (2006). The model organism as a system: integrating ’omics’ data sets. *Nature Reviews Molecular Cell Biology*.

[37] Seliger H (2007). Introduction array technology—an overview. *Methods in Molecular Biology*.

[38] Ibrahim AFM, Hedley PE, Cardle L (2005). A comparative analysis of transcript abundance using SAGE and Affymetrix arrays. *Functional and Integrative Genomics*.

[39] Tuteja R, Tuteja N (2004). Serial analysis of gene expression: applications in malaria parasite, yeast, plant, and animal studies. *Journal of Biomedicine and Biotechnology*.

[40] Bertrand G, Coste J, Segarra C, Schved JF, Commes T, Marti J (2004). Use of serial analysis of gene expression (SAGE) technology reveals new granulocytic markers. *Journal of Immunological Methods*.

[41] Boon K, Edwards JB, Eberhart CG, Riggins GJ (2004). Identification of astrocytoma associated genes including cell surface markers. *BMC Cancer*.

[42] Yamamoto M, Wakatsuki T, Hada A, Ryo A (2001). Use of serial analysis of gene expression (SAGE) technology. *Journal of Immunological Methods*.

[43] Faaberg KS, Perlman S, Gallagher T, Snijder EJ (2008). Arterivirus structural proteins and assembly. *Nidoviruses*.

[44] López-Fuertes L, Campos E, Doménech N (2000). Porcine reproductive and respiratory syndrome (PRRS) virus down-modulates TNF-*α* production in infected macrophages. *Virus Research*.

[45] Lee SM, Kleiboeker SB (2005). Porcine arterivirus activates the NF-*κ*B pathway through I*κ*B degradation. *Virology*.

[46] Lee SM, Schommer SK, Kleiboeker SB (2004). Porcine reproductive and respiratory syndrome virus field isolates differ in in vitro interferon phenotypes. *Veterinary Immunology and Immunopathology*.

[47] Genini S, Delputte PL, Malinverni R (2008). Genome-wide transcriptional response of primary alveolar macrophages following infection with porcine reproductive and respiratory syndrome virus. *Journal of General Virology*.

[48] Murtaugh MP, Elam MR, Kakach LT (1995). Comparison of the structural protein coding sequences of the VR-2332 and Lelystad virus strains of the PRRS virus. *Archives of Virology*.

[49] Sprenger H, Meyer RG, Kaufmann A, Bußfeld D, Rischkowsky E, Gemsa D (1996). Selective induction of monocyte and not neurophil-attracting chemokines after influenza A virus infection. *Journal of Experimental Medicine*.

[50] Knoetig SM, Summerfield A, Spagnuolo-Weaver M, McCullough KC (1999). Immunopathogenesis of classical swine fever: role of monocytic cells. *Immunology*.

[51] Salazar-Mather TP, Hokeness KL (2006). Cytokine and chemokine networks: pathways to antiviral defense. *Current Topics in Microbiology and Immunology*.

[52] Snijder EJ, Meulenberg JJM (1998). The molecular biology of arteriviruses. *Journal of General Virology*.

[53] Hiscott J, Kwon H, Génin P (2001). Hostile takeovers: viral appropriation of the NF-*κ*B pathway. *Journal of Clinical Investigation*.

[54] Bansal V, Ochoa JB (2003). Arginine availability, arginase, and the immune response. *Current Opinion in Clinical Nutrition and Metabolic Care*.

[55] Chang CI, Liao JC, Kuo L (1998). Arginase modulates nitric oxide production in activated macrophages. *American Journal of Physiology*.

[56] Johann AM, Barra V, Kuhn AM, Weigert A, Von Knethen A, Brüne B (2007). Apoptotic cells induce arginase II in macrophages, thereby attenuating NO production. *FASEB Journal*.

[57] Chiou MT, Jeng CR, Chueh LL, Cheng CH, Pang VF (2000). Effects of porcine reproductive and respiratory syndrome virus (isolate tw91) on porcine alveolar macrophages in vitro. *Veterinary Microbiology*.

[58] Opriessnig T, Yu S, Gallup JM (2003). Effect of vaccination with selective bacterins on conventional pigs infected with type 2 porcine circovirus. *Veterinary Pathology*.

[59] Stakenborg T, Vicca J, Butaye P (2006). A multiplex PCR to identify porcine mycoplasmas present in broth cultures. *Veterinary Research Communications*.

[60] Allende R, Kutish GF, Laegreid W (2000). Mutations in the genome of porcine reproductive and respiratory syndrome virus responsible for the attenuation phenotype. *Archives of Virology*.

[61] Kim HS, Kwang J, Yoon IJ, Joo HS, Frey ML (1993). Enhanced replication of porcine reproductive and respiratory syndrome (PRRS) virus in a homogeneous subpopulation of MA-104 [MA104] cell line. *Archives of Virology*.

[62] Velculescu VE, Zhang L, Vogelstein B, Kinzler KW (1995). Serial analysis of gene expression. *Science*.

[63] Beißbarth T, Hyde L, Smyth GK (2004). Statistical modeling of sequencing errors in SAGE libraries. *Bioinformatics*.

[64] Madden SL, Wang CJ, Landes G (2000). Serial analysis of gene expression: from gene discovery to target identification. *Drug Discovery Today*.

[65] Keime C, Damiola F, Mouchiroud D, Duret L, Gandrillon O (2004). Identitag, a relational database for SAGE tag identification and interspecies comparison of SAGE libraries. *BMC Bioinformatics*.

[66] Romualdi C, Bortoluzzi S, D’Alessi F, Danieli GA (2003). IDEG6: a web tool for detection of differentially expressed genes in multiple tag sampling experiments. *Physiological Genomics*.

[67] Liu W, Saint DA (2002). Validation of a quantitative method for real time PCR kinetics. *Biochemical and Biophysical Research Communications*.

[68] Livak KJ, Schmittgen TD (2001). Analysis of relative gene expression data using real-time quantitative PCR and the 2^−ΔΔ^T method. *Methods*.

